# Crystal structures of (2*E*)-1-(3-bromo­thio­phen-2-yl)-3-(2-meth­oxy­phen­yl)prop-2-en-1-one and (2*E*)-1-(3-bromo­thio­phen-2-yl)-3-(3,4-di­meth­oxy­phen­yl)prop-2-en-1-one

**DOI:** 10.1107/S2056989015013420

**Published:** 2015-07-25

**Authors:** Vasant S. Naik, Venkataraya Shettigar, Tyler S. Berglin, Jillian S. Coburn, Jerry P. Jasinski, Hemmige S. Yathirajan

**Affiliations:** a Department of Physics, Government First Grade College, Kumta 581 343, India, Research and Development Centre, Bharathiar University, Coimbatore 641 046, India; b Department of Physics, Gokhale Centenary College, Ankola 581 314, India, Research and Development Centre, Bharathiar University, Coimbatore 641 046, India; c Department of Chemistry, Keene State College, 229 Main Street, Keene, NH 03435-2001, USA; d Department of Studies in Chemistry, University of Mysore, Manasagangotri, Mysuru 570 006, India

**Keywords:** crystal structure, bromo­thio­phene, meth­oxy­phenyl­prop-2-en-1-one, mol­ecular conformation, nearly coplanar mol­ecules, C—H⋯π inter­actions, π–π sacking inter­actions

## Abstract

In the mol­ecules of the title compounds, (2*E*)-1-(3-bromo-thio­phen-2-yl)-3-(2-meth­oxy­phen­yl)prop-2-en-1-one, C_14_H_11_BrO_2_S, (I), which crystallizes in the space group *P*-1 with four independent mol­ecules in the asymmetric unit (*Z*′ = 8), and (2*E*)-1-(3-bromo­thio­phen-2-yl)-3-(3,4-di­meth­oxy­phen­yl)prop-2-en-1-one, C_15_H_13_BrO_3_S, (II), which crystallizes with *Z*′ = 8 in the space group *I*2/*a*, the non-H atoms are nearly coplanar. The mol­ecules of (I) pack with inversion symmetry stacked diagonally along the *a-*axis direction. Weak C—H⋯Br intra­molecular inter­actions in each of the four mol­ecules in the asymmetric unit are observed. In (II), weak C—H⋯O, bifurcated three-center inter­molecular inter­actions forming dimers along with weak C—H⋯π and π–π stacking inter­actions are observed, linking the mol­ecules into sheets along [001]. A weak C—H⋯Br intra­molecular inter­action is also present. There are no classical hydrogen bonds present in either structure.

## Abstract 2

Two closely related related nearly coplanar mol­ecules of 1-(3-bromo­thio­phen-2-yl)-3-(meth­oxy­phen­yl)prop-2-en-1-ones exhibit different patterns of weak inter- or intra­molecular inter­actions and crystallize in different space groups.

## Chemical context   

Chalcones are known for their inter­esting pharmacological activities (Di Carlo *et al.*, 1999 ▸). A review on the bioactivities of chalcones has been published (Dimmock *et al.*, 1999 ▸). Chalcones and their heterocyclic analogs as potential anti­fungal chemotherapeutic agents have been reported (Opletalová & Sedivý, 1999 ▸). Chalcones and flavonoids as anti-tuberculosis agents are reported (Lin *et al.*, 2002 ▸). Also, chalcones are recognized material in the photonic industry because of their excellent blue-light transmittance and good crystallizability properties (Goto *et al.* 1991 ▸; Indira *et al.*, 2002 ▸; Sarojini *et al.*, 2006 ▸). 2-Acetyl-3-bromo­thio­phene is one of the well-known bio-active inter­mediates, and chalcones of 2-acetyl-3-bromo­thio­phene exhibit promising anti-inflammatory, analgesic and anti­bacterial activities (Ashalatha, *et al.* 2009 ▸).

**Figure 1:**
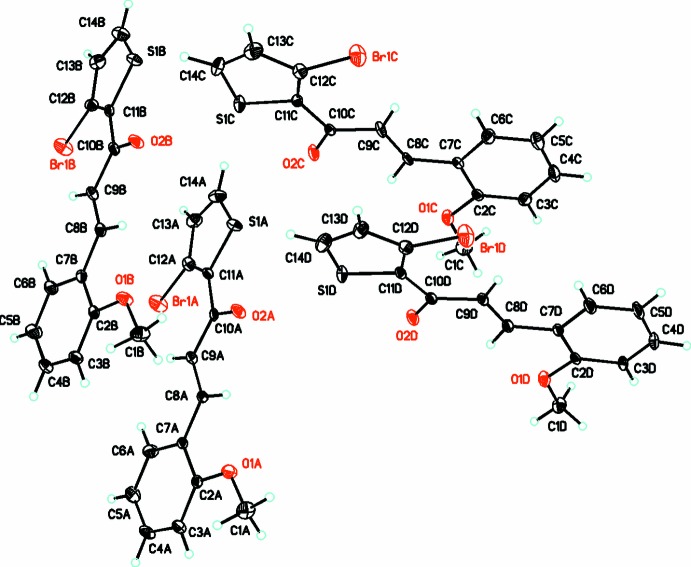
The mol­ecular structure of title compound (I), C_14_H_11_BrO_2_S, showing the atom-labelling scheme with 30% probability displacement ellipsoids.

**Figure 2:**
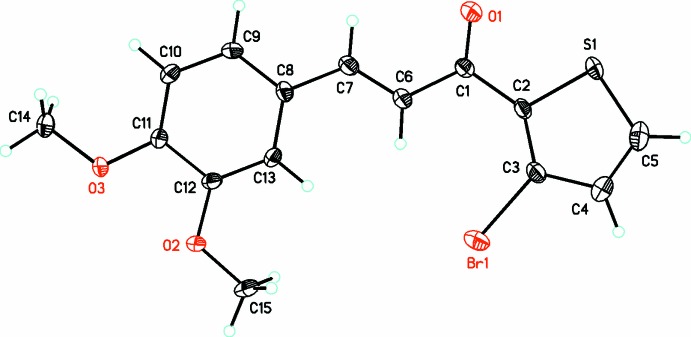
The mol­ecular structure of title compound (II), C_15_H_13_BrO_3_S, showing the atom-labelling scheme with 30% probability displacement ellipsoids.

Here we report the crystal structures of two new chalcones, namely (2*E*)-1-(3-bromo-2-thio­phen-2-yl)-3-(2-meth­oxy­phen­yl)prop-2-en-1-one, C_14_H_11_BrO_2_S, (I) (Fig. 1 ▸) and (2*E*)-1-(3-bromo-2-thio­phen-2-yl)-3-(3,4-di­meth­oxy­phen­yl)prop-2-en-1-one, C_15_H_13_BrO_3_S, (II) (Fig. 2 ▸). Compounds (I) and (II) are of the general type *P*C_3_H_2_O*R* and *Q*C_3_H_2_O*R* where *P* represents the 2-meth­oxy­phenyl unit in (I), *Q* represents the 3,4-dimeth­oxy unit in (II) and *R* the 3-bromo­[thio­phenyl unit in (I) and (II). The mol­ecular constitutions of compounds (I) and (II) differ only in the number of the meth­oxy­phenyl substituents, whereby (I) contains only one, *P* unit, at an *ortho* position, and (II) contains two, *Q*, units at the *meta* and *para* positions of the phenyl ring.
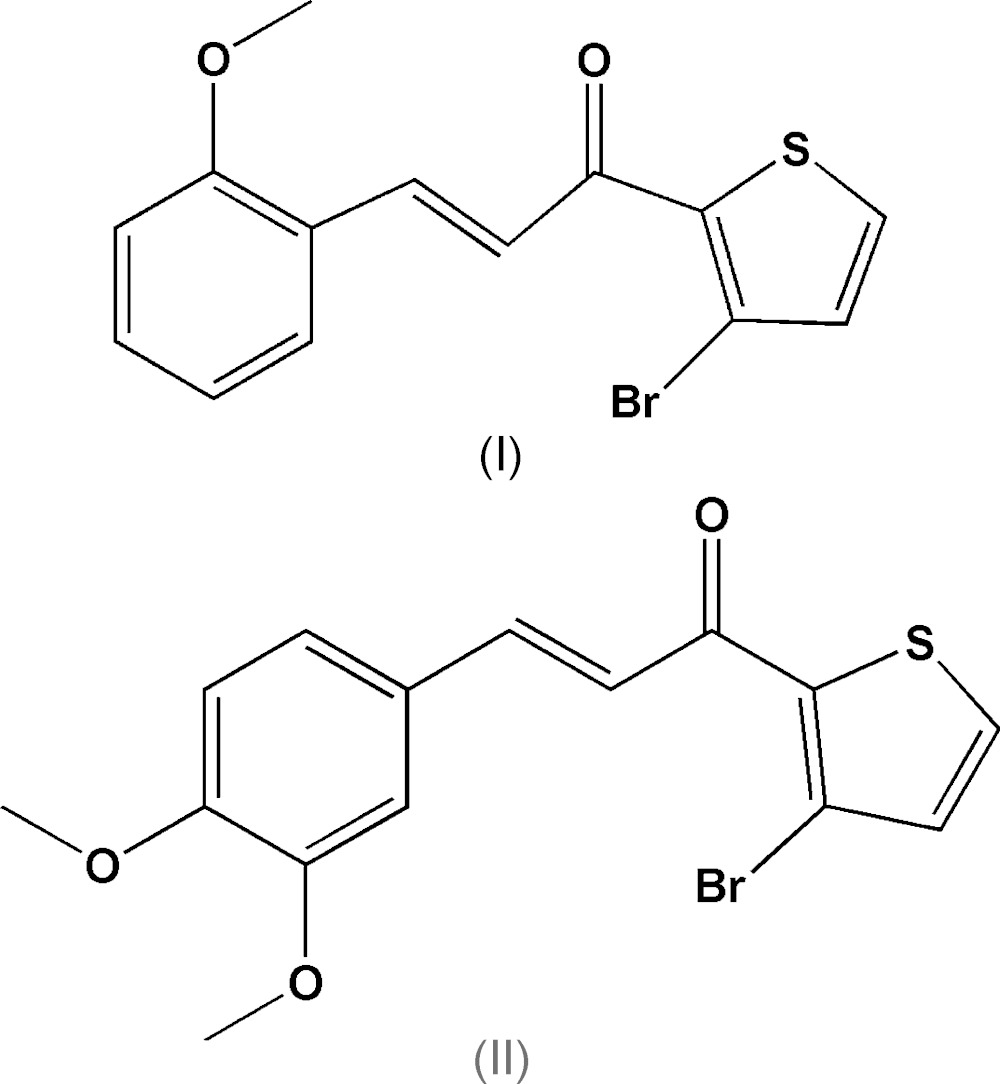


## Structural commentary   

The structure of C_14_H_11_BrO_2_S, (I), has triclinic (*P* 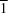) symmetry, while in (II), C_15_H_13_BrO_3_S, it crystallizes in the monoclinic, *I*2/*a* space group. In (I), four independent mol­ecules (*A*, *B*, *C*, *D*) crystallize in the asymmetric unit (*Z*′ = 8) (Fig. 1 ▸), while only one mol­ecule (*Z*′ = 8) is present in (II) (Fig. 2 ▸). A search for possible additional crystallographic symmetry or pseudosymmetry in compound (II) (Spek, 2009 ▸) produced none, while in compound (I) there was indication of the possibility of either *P* 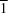 symmetry with the *a-*axis halved or the presence of *C*2/*c* symmetry. Structural solution of the structure in the C*2*/*c* space group after transforming the axes in *PLATON* gave a negative result, confirming the (*P* 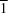) symmetry assignment. Refinement of the structure with two independent mol­ecules in the asymmetry unit rather than four also gave a negative result, even though the coordinates for the *A*/*B* and *C*/*D* pairs of mol­ecules are related by translation of 0.5 along the *a* axis, displaying pseudo symmetry which gave *B* alerts in *checkCIF* even after many cycles of refinement.

In the mol­ecular structures of both compounds, (I) and (II), the non-H atoms are almost coplanar, as shown by their relevant torsional and dihedral angles (Table 1 ▸). In (I), the mean plane of the keto group is twisted slightly out of plane with that of the thio­phene ring in the range of 3–4° and with torsion angles in the range of 174–176° in each of the four mol­ecules (Table 1 ▸). The dihedral angle between the mean planes of the phenyl and thio­phene rings are in the range of 10–11°. In (II), the mean plane of the keto group is twisted slightly out of plane with that of the thio­phene ring by 0.9 (9)°, with a torsion angle of −178.2 (6)°, and a dihedral angle between the mean planes of the phenyl and thio­phene rings of 8.4 (2)°. In both compounds, bond lengths and angles are in normal ranges (Allen *et al.*, 2002 ▸).

## Supra­molecular features   

**Figure 3:**
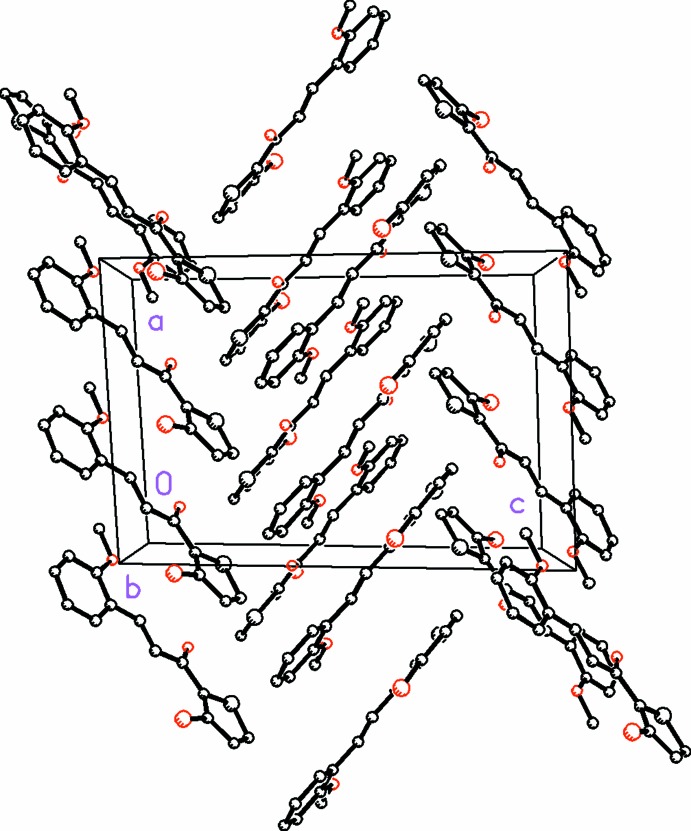
The mol­ecular packing for compound (I), viewed along the *a* axis, showing zigzag layers in (010). H atoms not involved in hydrogen bonding and weak inter­molecular inter­actions have been omitted for clarity.

**Figure 4:**
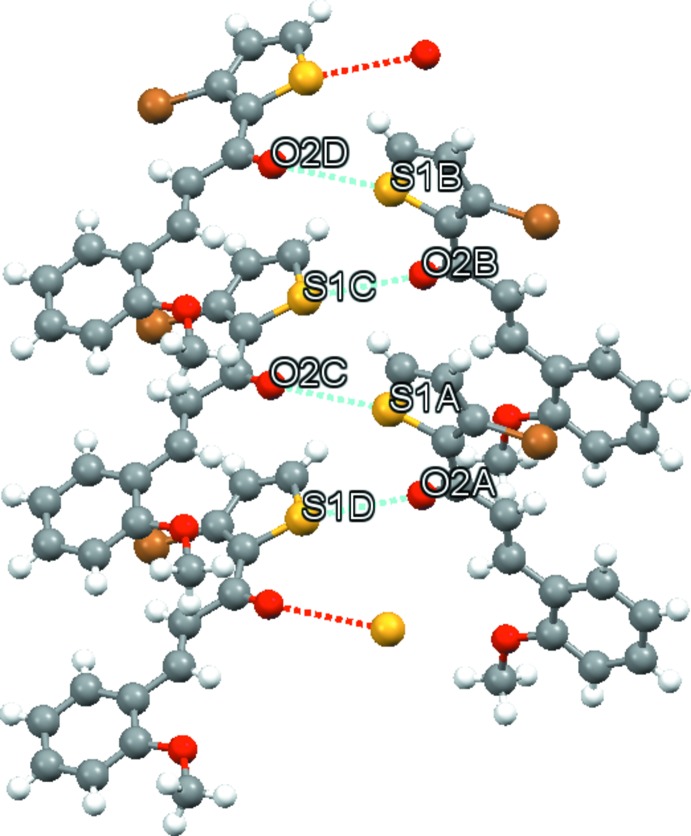
A view of the asymmetric unit in (I), with dashed lines showing short O⋯S inter­molecular contacts between each mol­ecule pair [S1*D⋯*O2*A* = 3.14 (1), O2*C*⋯S1*A* = 3.13 (5), S1*C*⋯O2*B* = 3.13 (8), O2*D*⋯S1*B* = 3.14 (1) Å].

**Figure 5:**
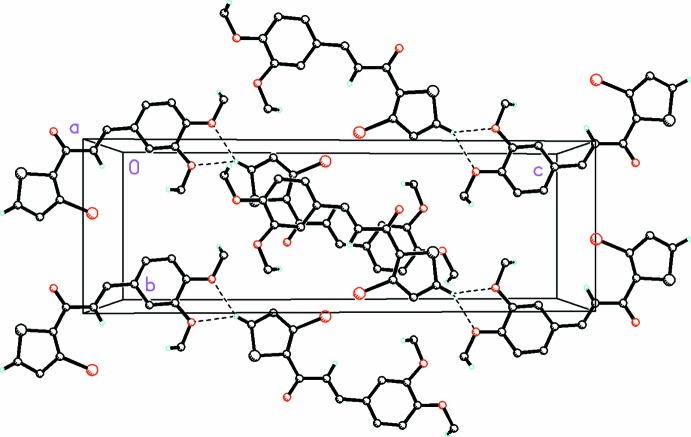
The mol­ecular packing for compound (II), viewed along the *a* axis. Dashed lines indicate weak C—H⋯O inter­molecular inter­actions displaying bifurcated three-center character, forming dimers in layers along [001]. H atoms not involved in hydrogen bonding or weak inter­molecular inter­actions have been omitted for clarity.

The presence of weak C—H⋯Br intra­molecular bonds (Table 2 ▸) and absence of any direction-specific weak inter­molecular inter­actions in (I) in contrast to the presence of a variety of weak C—H⋯O, C—H⋯π and π–π inter­molecular inter­actions in (II) (Table 3 ▸) is suggestive of this type of support in describing the slight differences in planarity of the mol­ecules that is observed between the two compounds. The mol­ecules in (I) pack in zigzag layers in (010) (Fig. 3 ▸). Within the asymmetric unit, short O—S inter­molecular contacts aligned between each mol­ecule pair [S1*D⋯*O2*A* = 3.14 (1), O2*C*⋯S1*A* = 3.13 (5), S1*C*⋯O2*B* = 3.13 (8), O2*D*⋯S1*B* = 3.14 (1) Å] are also observed (Fig. 4 ▸). In (II), weak C5—H5⋯O2 and C5—H5⋯O3 inter­actions display bifurcated three-center character, forming dimers in layers along [001] (Fig. 5 ▸). Additionally, π–π stacking inter­actions occur between the thio­phene (S/C2–C5) and phenyl rings (C8–C13) with a ring centroid separation of 3.840 (3)Å, and a shortest perpendicular distance from the centroid of one ring to the plane of the other of 3.454 (2) Å.

## Database survey   

**Figure 6:**
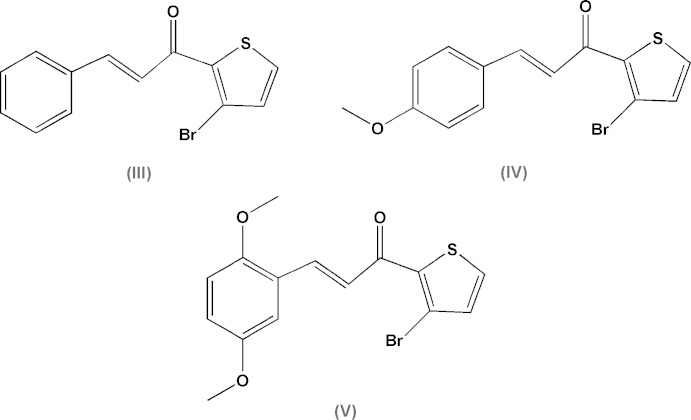
Compounds (III), (IV) and (V).

A search of the Cambridge Structural Database (Version 5.36, last update February 2015: Allen 2002 ▸) revealed three closely related (3-bromo-2-thio­phen-2-yl)-3-(di­meth­oxy­phen­yl)prop-2-en-1-one types of compounds similar to the title compounds in this study and will be referred to as (III) (2*E*)-1-(3-bromo­thien-2-yl)-3-phenyl­prop-2-en-1-one (Butcher *et al.*, 2007*d* ▸), (IV) (2*E*)-1-(3-bromo-2-thio­phen-2-yl)-3-(4-meth­oxy­phen­yl)prop-2-en-1-one (Harrison *et al.*, 2006 ▸) and (V) (2*E*)-1-(3-bromo-2-thio­phen-2-yl)-3-(2,5-di­meth­oxy­phen­yl)prop-2-en-1-one (Yathirajan *et al.*, 2006 ▸) for structural comparisons (Fig. 6 ▸).

The crystal structures of some other related chalcones, *viz*., (2*E*)-1-(3-bromo-2-thio­phen-2-yl)-3-(4-meth­oxy-2,3,6-trimethyl­phen­yl)prop-2-en-1-one (Yathirajan *et al.*, 2006*a* ▸), (2*E*)-1-(3-bromo-2-thio­phen-2-yl)-3-(4,5-dimeth­oxy-2-nitrophen­yl)prop-2-en-1-one (Yathirajan *et al.*, 2006*b* ▸), (2*E*)-1-(3-bromo-2-thien­yl)-3-(2,5-di­meth­oxy­phen­yl)prop-2-en-1-one (Yathirajan *et al.*, 2006*c* ▸), 1-(3-bromo-2-thien­yl)-3-[4-(di­methyl­amino)­phen­yl]prop-2-en-1-one (Butcher *et al.*, 2007*a* ▸), 1-(3-bromo-2-thien­yl)-3-(4-but­oxyphen­yl)prop-2-en-1-one (Butcher *et al.*, 2007*b* ▸) and 1-(3-bromo-2-thien­yl)-3-(6-meth­oxy-2-naphth­yl)prop-2-en-1-one (Butcher *et al.*, 2007*c* ▸) have also been reported.

Compound (IV) is structurally similar to (I) with the only difference occurring in the *P* unit with the meth­oxy group now in the *para* position on the phenyl ring. Compound (V) is structurally similar to (II) with the *Q* unit now containing the two meth­oxy groups at the *ortho* and *meta* positions of the phenyl ring, Compound (III), which crystallized with two independent mol­ecules in the asymmetric unit, is structurally similar to both (I) and (II) except with no meth­oxy groups on the phenyl ring. The *R* units are structurally identical in all five compounds described here.

A comparison of the supra­molecular features of the title compounds (Table 4 ▸) suggests that the presence or absence of direction-specific weak inter­molecular inter­actions plays a role in their influence on the small differences in planarity observed and supported by similar types of inter­actions in closely related compounds. No classical hydrogen bonds are observed in any of the five compounds. All five compounds do display a similar weak C—H⋯Br intra­molecular inter­action. In (I) and (III) only weak C—H⋯π inter­molecular inter­actions are observed, while in (IV) only weak C—H⋯O inter­molecular inter­actions are present.

In (II), the weak C5—H5⋯O2 and C5—H5⋯O3 inter­actions display bifurcated three-center character, forming dimers in layers along [001]. Additionally, C—H⋯π and π–π stacking inter­actions (Table 4 ▸) are observed, which help pack the mol­ecules into a two-dimensional network (Fig. 4 ▸). In (V), weak C—H⋯O also form bifurcated three-center character in a similar fashion to (II).

## Synthesis and crystallization   

For crystals (I) and (II), the following procedure was used. A solution of 3-bromo-2-acetyl­thio­phene (2.05 g, 0.01 mol) in methanol (20 ml) was mixed with 2-meth­oxy­benzaldehyde (1.36 g, 0.01 mol) for crystal (I) and 3,4-di­meth­oxy­benzaldehyde (1.66 g, 0.01 mol) for crystal (II) in methanol (20 ml) in the presence of NaOH (5 ml, 30%) at 283 K. After stirring for four h, the contents of the flask were poured into ice-cold water (250 ml). The resulting crude solid was collected by filtration and dried in a hot-air oven at 323 K. A supersaturated solution was obtained by dissolving the sample in acetone at ambient temperature. The prepared solution was filtered, warmed slightly and allowed to evaporate slowly at room temperature. After several days X-ray quality crystals were obtained by the slow the evaporation technique, m.p.: 367 K for (I) and 405 K for (II).

## Refinement   

Crystal data, data collection and structure refinement details are summarized in Table 5 ▸. In both (I) and (II), all H atoms were located in difference maps. The H atoms bonded to C atoms were then treated as riding atoms in geometrically idealized positions with C—H distances 0.95 Å (aromatic and hetero-aromatic) or 0.98 Å (CH_3_) and with *U*_iso_(H) = k*U*_eq_(C), where k = 1.5 for the methyl groups, which were permitted to rotate but not to tilt, and 1.2 for all other H atoms bonded to C atoms. The maximum residual electron density peaks of 1.17 and −0.82 Å^3^, for (I), were located at 0.94 and 0.84 Å from Br1, respectively. For (II), the maximum residual electron density peaks of 3.38 and −2.20 Å^3^ were located at 0.94 and 0.84 Å from Br1.

## Supplementary Material

Crystal structure: contains datablock(s) Global, I, II. DOI: 10.1107/S2056989015013420/hg5448sup1.cif

Structure factors: contains datablock(s) I. DOI: 10.1107/S2056989015013420/hg5448Isup2.hkl

Structure factors: contains datablock(s) II. DOI: 10.1107/S2056989015013420/hg5448IIsup3.hkl

CCDC references: 1412491, 1412490

Additional supporting information:  crystallographic information; 3D view; checkCIF report

## Figures and Tables

**Table 1:** Selected torsional and dihedral angles (°) for compounds (I), (II), (III), (IV) and (V) Dihedral 1 represents the dihedral angle between the mean planes of the phenyl and thio­phene rings, Dihedral 2 represents the dihedral angle between the mean planes of the thio­phene ring and the keto unit, and Dihedral 3 represents the dihedral angle between the mean planes of the phenyl ring and the keto unit.

Parameter	(I)	(II)	(III)	(IV)	(V)
C12*A*—C11*A*—C10*A*—O2*A*	174.3 (5)				
C12*B*—C11*B*—C10*B*—O2*B*	175.8 (5)				
C12*C*—C11*C*—C10*C*—O2*C*	174.3 (5)				
C12*D*—C11*D*—C10*D*—O2*D*	176.5 (5)				
C3—C2—C1—O1		−178.2 (6)			
C3*A*—C4*A*—C5*A*—O1*A*			−176.5 (7)		
C3*B*—C4*B*—C5*B*—O1*B*			178.2 (8)		
C3—C4—C5—O1				161.0 (3)	
C2—C1—C5—O5					3.3 (8)
Dihedral 1	11.3 (6)				
	10.9 (6)				
	11.3 (6)				
	11.1 (1)				
		8.4 (2)			
			4.9 (7)		
			12.2 (4)		
				19.5 (7)	
					7.1 (8)
Dihedral 2	4.1 (4)				
	3.4 (9)				
	3.0 (3)				
	3.3 (2)				
		0.9 (9)			
			2.8 (2)		
			5.1 (1)		
				18.6 (3)	
					4.0 (9)
Dihedral 3	7.4 (3)				
	7.7 (5)				
	7.3 (1)				
	7.6 (6)				
		9.1 (1)			
			3.8 (2)		
			9.8 (9)		
				10.2 (0)	
					3.8 (7)

**Table 2:** Hydrogen-bond geometry (Å, °) for (I)

*D*—H⋯*A*	*D*—H	H⋯*A*	*D*⋯*A*	*D*—H⋯*A*
C9*C*—H9*C*⋯Br1*C*	0.95	2.68	3.401 (5)	133
C9*D*—H9*D*⋯Br1*D*	0.95	2.69	3.405 (4)	133
C9*A*—H9*A*⋯Br1*A*	0.95	2.69	3.398 (5)	132
C9*B*—H9*B*⋯Br1*B*	0.95	2.68	3.401 (5)	133

**Table 3:** Hydrogen-bond geometry (Å, °) for (II)

*D*—H⋯*A*	*D*—H	H⋯*A*	*D*⋯*A*	*D*—H⋯*A*
C5—H5⋯O2^i^	0.95	2.52	3.301 (6)	140
C5—H5⋯O3^i^	0.95	2.45	3.291 (6)	148
C6—H6⋯Br1	0.95	2.59	3.361 (5)	139
C14—H14*A*⋯O1^ii^	0.98	2.59	3.495 (6)	154

Symmetry codes: (i) 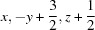; (ii) 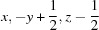.

**Table 4:** Hydrogen bonds and short inter­molecular contacts (Å, °) for compounds (I), (II), (III), (IV) and (V) *Cg*2(I) represents the centroid of the ring C2*A*–C7*A*, *Cg*4(I) represents the centroid of the ring C2*B*–C7*B*, *Cg*6(I) represents the centroid of the ring C2*C*–C7*C*, *Cg*8(I) represents the centroid of the ring C2*D*–C7*D*, *Cg*1(II) represents the centroid of the ring S1/C2–C5, *Cg*1(III) represents the centroid of the ring S1*A*/C1*A*–C4*A*, *Cg*2(III) represents the centroid of the ring C8*A*–C13*A*, *Cg*3(III) represents the centroid of the ring S1*B*/C1*B*–C4*B*.

Compound	*D*—H⋯*A*	*D*—H	H⋯*A*	*D*⋯A	*D*—H⋯*A*
(I)	C9*A*—H9*A*⋯Br1*A*	0.95	2.68	3.400 (5)	132
	C9*B*—H9*B*⋯Br1*B*	0.95	2.68	3.401 (5)	132
	C9*C*—H9*C*⋯Br1*C*	0.95	2.68	3.400 (5)	133
	C9*D*—H9*D*⋯Br1*D*	0.95	2.68	3.405 (4)	133
	C13*A*—H13*A*⋯*Cg*8^i^		2.96	3.678 (6)	134
	C13*B*—H13*B*⋯*Cg*6^ii^		2.96	3.666 (6)	132
	C13*C*—H13*C*⋯*Cg*4^iii^		2.95	3.667 (6)	133
	C13*D*—H13*D*⋯*Cg*2^iv^		2.94	3.664 (5)	134
(II)	C5—H5⋯O2^v^	0.95	2.52	3.301 (6)	140
	C5—H5⋯O3^vi^	0.95	2.45	3.291 (6)	147
	C14—H14*A*⋯O1^vii^	0.98	2.59	3.495 (6)	154
	C6—H6⋯Br1	0.95	2.59	3.361 (5)	139
	C15—H15*B*⋯*Cg*1(II)^viii^		2.98	3.734 (7)	135
(III)	C6*A*—H6*AA*⋯Br1*A*	0.95	2.61	3.1367 (3)	137
	C6*B*—H6*BA*⋯Br1*B*	0.95	2.68	3.421 (8)	135
	C1*A*—H1*A*⋯*Cg*2(III)^ix^		2.87	3.566 (8)	131
	C10*A*—H10*A*⋯*Cg*1(III)^*x*^		3.00	3.668 (8)	129
	C10*B*—H10*B*⋯*Cg*3(III)^xi^		2.92	3.659 (8)	135
(IV)	C1—H1⋯O2^xii^	0.95	2.54	3.457 (3)	162
	C14—H14*B*⋯O1^xiii^	0.98	2.45	3.300 (4)	145
	C6—H6⋯Br1	0.95	2.73	3.410 (3)	129
(V)	C4—H4⋯O5^xiv^	0.96	2.37	3.296 (3)	164
	C17—H17⋯O5^xv^	0.98	2.41	3.331 (9)	157
	C6—H6⋯Br1	0.95	2.68	3.394 (7)	133

Symmetry codes: (i) 1 − *x*, 1 − *y*, −*z*; (ii) −*x*, 1 − *y*, −*z*; (iii) −*x*, 1 − *y*, 1 − *z*; (iv) 1 − *x*, 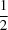 + *y*, 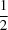 − *z*; (v) *x*, 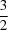 − *y*, 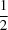 + *z*; (vi) *x*, 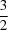 − *y*, 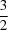 + *z*; (vii) *x*, 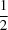 − *y*, −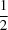 + *z*; (viii) 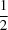 − *x*, −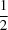 + *y*, 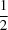 − *z*; (ix) 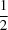 + *x*, − *y*, *z*; (*x*) −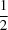 − *x*, 1 − *y*, *z*; (xi) 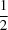 + *x*, −*y*, *z*; (xii) *x*, *y*, −1 + *z*; (xiii) −*x*, 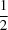 + *y*, 1 − *z*; (xiv) 3 − *x*, 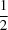 + *y*, 2 − *z*; (xv) 1 − *x*, −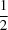 + *y*, 2 − *z*.

**Table 5:** Experimental details

	(I)	(II)
Crystal data		
Chemical formula	C_14_H_11_BrO_2_S	C_15_H_13_BrO_3_S
*M*_r_	323.20	353.22
Crystal system, space group	Triclinic, *P* 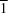	Monoclinic, *I*2/*a*
Temperature (K)	173	173
*a*, *b*, *c* (Å)	11.2517 (4), 14.5397 (6), 16.7857 (6)	13.4748 (7), 8.3853 (3), 25.0214 (9)
α, β, γ (°)	76.561 (3), 89.989 (3), 78.836 (3)	90, 93.957 (4), 90
*V* (Å^3^)	2617.44 (17)	2820.4 (2)
*Z*	8	8
Radiation type	Cu *K*α	Cu *K*α
μ (mm^−1^)	5.70	5.40
Crystal size (mm)	0.49 × 0.44 × 0.28	0.32 × 0.28 × 0.22

Data collection		
Diffractometer	Agilent Eos Gemini	Agilent Eos Gemini
Absorption correction	Multi-scan (*CrysAlis PRO*; Agilent, 2014 ▸)	Multi-scan (*CrysAlis PRO*; Agilent, 2014 ▸)
*T*_min_, *T*_max_	0.353, 1.000	0.726, 1.000
No. of measured, independent and observed [*I* > 2σ(*I*)] reflections	19842, 9990, 4573	5523, 2690, 2399
*R*_int_	0.037	0.026
(sin θ/λ)_max_ (Å^−1^)	0.614	0.615

Refinement		
*R*[*F* ^2^ > 2σ(*F* ^2^)], *wR*(*F* ^2^), *S*	0.047, 0.168, 1.01	0.074, 0.187, 1.04
No. of reflections	9990	2690
No. of parameters	653	183
H-atom treatment	H-atom parameters constrained	H-atom parameters constrained
(Δ/σ)_max_	0.148	< 0.001
Δρ_max_, Δρ_min_ (e Å^−3^)	1.17, −0.82	3.38, −2.20

Computer programs: *CrysAlis PRO* and *CrysAlis RED* (Agilent, 2014 ▸), *SHELXT* (Sheldrick, 2015*a* ▸), *SHELXL2014* (Sheldrick, 2015*b* ▸) and *OLEX2* (Dolomanov *et al.*, 2009 ▸).

## supplementary crystallographic information

### (I) (2E)-1-(3-Bromothiophen-2-yl)-3-(2-methoxyphenyl)prop-2-en-1-one Crystal data

**Table:**

C_14_H_11_BrO_2_S	*Z* = 8
*M**_r_* = 323.20	*F*(000) = 1296
Triclinic, *P*1	*D*_x_ = 1.640 Mg m^−^^3^
*a* = 11.2517 (4) Å	Cu *K*α radiation, λ = 1.54184 Å
*b* = 14.5397 (6) Å	Cell parameters from 5217 reflections
*c* = 16.7857 (6) Å	θ = 4.6–70.9°
α = 76.561 (3)°	µ = 5.70 mm^−^^1^
β = 89.989 (3)°	*T* = 173 K
γ = 78.836 (3)°	Irregular, colourless
*V* = 2617.44 (17) Å^3^	0.49 × 0.44 × 0.28 mm

### (I) (2E)-1-(3-Bromothiophen-2-yl)-3-(2-methoxyphenyl)prop-2-en-1-one Data collection

**Table:**

Agilent Eos Gemini diffractometer	9990 independent reflections
Radiation source: Enhance (Cu) X-ray Source	4573 reflections with *I* > 2σ(*I*)
Detector resolution: 16.0416 pixels mm^-1^	*R*_int_ = 0.037
ω scans	θ_max_ = 71.3°, θ_min_ = 4.0°
Absorption correction: multi-scan (CrysAlis PRO; Agilent, 2014)	*h* = −13→8
*T*_min_ = 0.353, *T*_max_ = 1.000	*k* = −17→17
19842 measured reflections	*l* = −20→20

### (I) (2E)-1-(3-Bromothiophen-2-yl)-3-(2-methoxyphenyl)prop-2-en-1-one Refinement

**Table:**

Refinement on *F*^2^	Primary atom site location: structure-invariant direct methods
Least-squares matrix: full	Hydrogen site location: inferred from neighbouring sites
*R*[*F*^2^ > 2σ(*F*^2^)] = 0.047	H-atom parameters constrained
*wR*(*F*^2^) = 0.168	*w* = 1/[σ^2^(*F*_o_^2^) + (0.0832*P*)^2^] where *P* = (*F*_o_^2^ + 2*F*_c_^2^)/3
*S* = 1.01	(Δ/σ)_max_ = 0.148
9990 reflections	Δρ_max_ = 1.17 e Å^−^^3^
653 parameters	Δρ_min_ = −0.82 e Å^−^^3^
0 restraints	

### (I) (2E)-1-(3-Bromothiophen-2-yl)-3-(2-methoxyphenyl)prop-2-en-1-one Special details

**Table:**

Experimental. Absorption correction: CrysAlisPro, Agilent Technologies, Version 1.171.37.31 (release 14-01-2014 CrysAlis171 .NET) (compiled Jan 14 2014,18:38:05) Empirical absorption correction using spherical harmonics, implemented in SCALE3 ABSPACK scaling algorithm.
Geometry. All e.s.d.'s (except the e.s.d. in the dihedral angle between two l.s. planes) are estimated using the full covariance matrix. The cell e.s.d.'s are taken into account individually in the estimation of e.s.d.'s in distances, angles and torsion angles; correlations between e.s.d.'s in cell parameters are only used when they are defined by crystal symmetry. An approximate (isotropic) treatment of cell e.s.d.'s is used for estimating e.s.d.'s involving l.s. planes.

### (I) (2E)-1-(3-Bromothiophen-2-yl)-3-(2-methoxyphenyl)prop-2-en-1-one Fractional atomic coordinates and isotropic or equivalent isotropic displacement parameters (Å^2^)

**Table:**

	*x*	*y*	*z*	*U*_iso_*/*U*_eq_	
Br1C	−0.03752 (6)	0.89566 (4)	0.11911 (4)	0.04215 (18)	
S1C	−0.02881 (12)	0.58646 (10)	0.21914 (8)	0.0281 (3)	
O1C	0.4824 (3)	0.6319 (3)	−0.0458 (2)	0.0298 (9)	
O2C	0.1708 (3)	0.5722 (3)	0.1207 (2)	0.0303 (8)	
C1C	0.5883 (5)	0.5823 (4)	−0.0767 (4)	0.0368 (14)	
H1CA	0.5816	0.5987	−0.1368	0.055*	
H1CB	0.5953	0.5125	−0.0562	0.055*	
H1CC	0.6603	0.6017	−0.0583	0.055*	
C2C	0.4461 (4)	0.7277 (4)	−0.0760 (3)	0.0198 (10)	
C3C	0.5054 (4)	0.7849 (4)	−0.1346 (3)	0.0268 (11)	
H3C	0.5779	0.7567	−0.1561	0.032*	
C4C	0.4596 (5)	0.8819 (4)	−0.1616 (3)	0.0319 (12)	
H4C	0.5019	0.9202	−0.2008	0.038*	
C5C	0.3544 (5)	0.9244 (4)	−0.1332 (4)	0.0390 (14)	
H5C	0.3224	0.9914	−0.1534	0.047*	
C6C	0.2948 (5)	0.8678 (4)	−0.0741 (3)	0.0306 (12)	
H6C	0.2225	0.8976	−0.0534	0.037*	
C7C	0.3374 (4)	0.7697 (3)	−0.0445 (3)	0.0192 (10)	
C8C	0.2771 (4)	0.7080 (3)	0.0168 (3)	0.0211 (10)	
H8C	0.3168	0.6422	0.0335	0.025*	
C9C	0.1732 (4)	0.7324 (3)	0.0525 (3)	0.0231 (10)	
H9C	0.1307	0.7975	0.0403	0.028*	
C10C	0.1253 (4)	0.6578 (4)	0.1105 (3)	0.0187 (10)	
C11C	0.0180 (4)	0.6843 (4)	0.1582 (3)	0.0189 (10)	
C12C	−0.0524 (5)	0.7689 (4)	0.1692 (3)	0.0259 (11)	
C13C	−0.1438 (5)	0.7531 (5)	0.2268 (3)	0.0361 (14)	
H13C	−0.2000	0.8035	0.2416	0.043*	
C14C	−0.1415 (5)	0.6582 (4)	0.2579 (3)	0.0303 (12)	
H14C	−0.1961	0.6343	0.2968	0.036*	
Br1D	0.46260 (6)	0.89569 (4)	0.11921 (4)	0.04200 (18)	
S1D	0.47162 (12)	0.58643 (10)	0.21926 (8)	0.0272 (3)	
O1D	0.9843 (3)	0.6313 (3)	−0.0454 (2)	0.0300 (9)	
O2D	0.6696 (3)	0.5728 (3)	0.1210 (2)	0.0301 (8)	
C1D	1.0906 (4)	0.5823 (4)	−0.0765 (3)	0.0311 (12)	
H1DA	1.0727	0.5782	−0.1325	0.047*	
H1DB	1.1159	0.5171	−0.0415	0.047*	
H1DC	1.1561	0.6181	−0.0767	0.047*	
C2D	0.9458 (4)	0.7278 (4)	−0.0761 (3)	0.0187 (10)	
C3D	1.0067 (4)	0.7839 (4)	−0.1352 (3)	0.0267 (11)	
H3D	1.0781	0.7552	−0.1574	0.032*	
C4D	0.9610 (5)	0.8821 (4)	−0.1606 (3)	0.0355 (14)	
H4D	1.0044	0.9214	−0.1982	0.043*	
C5D	0.8523 (5)	0.9244 (4)	−0.1322 (3)	0.0357 (13)	
H5D	0.8193	0.9910	−0.1533	0.043*	
C6D	0.7927 (5)	0.8694 (4)	−0.0735 (3)	0.0298 (12)	
H6D	0.7204	0.8990	−0.0528	0.036*	
C7D	0.8376 (4)	0.7703 (3)	−0.0440 (3)	0.0193 (10)	
C8D	0.7773 (4)	0.7092 (4)	0.0175 (3)	0.0215 (10)	
H8D	0.8174	0.6435	0.0348	0.026*	
C9D	0.6745 (4)	0.7328 (3)	0.0525 (3)	0.0196 (9)	
H9D	0.6321	0.7980	0.0405	0.024*	
C10D	0.6254 (4)	0.6576 (4)	0.1104 (3)	0.0191 (10)	
C11D	0.5194 (4)	0.6847 (3)	0.1587 (3)	0.0184 (9)	
C12D	0.4472 (4)	0.7684 (4)	0.1688 (3)	0.0236 (10)	
C13D	0.3574 (4)	0.7535 (4)	0.2272 (3)	0.0303 (12)	
H13D	0.3014	0.8039	0.2421	0.036*	
C14D	0.3616 (5)	0.6586 (5)	0.2588 (4)	0.0361 (14)	
H14D	0.3087	0.6346	0.2990	0.043*	
Br1A	0.41043 (6)	0.10433 (4)	0.38081 (4)	0.04188 (19)	
S1A	0.26463 (11)	0.41362 (9)	0.28077 (7)	0.0270 (3)	
O1A	0.7996 (3)	0.3677 (3)	0.5461 (2)	0.0306 (9)	
O2A	0.4566 (3)	0.4277 (3)	0.3799 (2)	0.0295 (8)	
C1A	0.8804 (5)	0.4168 (4)	0.5775 (3)	0.0350 (13)	
H1AA	0.8602	0.4858	0.5509	0.052*	
H1AB	0.9640	0.3904	0.5665	0.052*	
H1AC	0.8725	0.4080	0.6368	0.052*	
C2A	0.8093 (4)	0.2730 (3)	0.5760 (3)	0.0198 (10)	
C3A	0.8983 (5)	0.2159 (4)	0.6342 (3)	0.0290 (12)	
H3A	0.9572	0.2442	0.6550	0.035*	
C4A	0.9009 (5)	0.1179 (4)	0.6616 (3)	0.0349 (14)	
H4A	0.9623	0.0795	0.7009	0.042*	
C5A	0.8147 (5)	0.0747 (4)	0.6324 (4)	0.0365 (13)	
H5A	0.8160	0.0078	0.6523	0.044*	
C6A	0.7271 (4)	0.1315 (4)	0.5736 (3)	0.0309 (13)	
H6A	0.6690	0.1025	0.5528	0.037*	
C7A	0.7227 (4)	0.2300 (3)	0.5443 (3)	0.0172 (9)	
C8A	0.6313 (4)	0.2924 (3)	0.4822 (3)	0.0194 (10)	
H8A	0.6386	0.3580	0.4645	0.023*	
C9A	0.5392 (4)	0.2671 (3)	0.4481 (3)	0.0203 (10)	
H9A	0.5292	0.2020	0.4610	0.024*	
C10A	0.4538 (4)	0.3418 (3)	0.3902 (3)	0.0188 (9)	
C11A	0.3599 (4)	0.3155 (3)	0.3419 (3)	0.0186 (9)	
C12A	0.3308 (4)	0.2328 (4)	0.3300 (3)	0.0246 (11)	
C13A	0.2337 (5)	0.2469 (4)	0.2734 (3)	0.0315 (12)	
H13A	0.2033	0.1963	0.2585	0.038*	
C14A	0.1897 (5)	0.3403 (4)	0.2432 (4)	0.0352 (14)	
H14A	0.1229	0.3640	0.2044	0.042*	
Br1B	−0.08962 (6)	0.10432 (4)	0.38073 (4)	0.04162 (19)	
S1B	−0.23554 (11)	0.41352 (10)	0.28088 (7)	0.0276 (3)	
O1B	0.2986 (3)	0.3684 (3)	0.5458 (2)	0.0301 (9)	
O2B	−0.0437 (3)	0.4276 (3)	0.3792 (2)	0.0294 (8)	
C1B	0.3811 (4)	0.4181 (4)	0.5759 (3)	0.0345 (13)	
H1BA	0.4638	0.3934	0.5619	0.052*	
H1BB	0.3773	0.4075	0.6356	0.052*	
H1BC	0.3584	0.4874	0.5507	0.052*	
C2B	0.3094 (4)	0.2722 (4)	0.5759 (3)	0.0201 (10)	
C3B	0.3978 (4)	0.2151 (4)	0.6341 (3)	0.0268 (11)	
H3B	0.4570	0.2432	0.6548	0.032*	
C4B	0.3999 (5)	0.1186 (4)	0.6617 (3)	0.0353 (14)	
H4B	0.4599	0.0806	0.7021	0.042*	
C5B	0.3158 (5)	0.0750 (4)	0.6318 (4)	0.0385 (14)	
H5B	0.3189	0.0077	0.6507	0.046*	
C6B	0.2273 (4)	0.1314 (4)	0.5738 (3)	0.0293 (12)	
H6B	0.1688	0.1022	0.5538	0.035*	
C7B	0.2222 (4)	0.2305 (3)	0.5441 (3)	0.0199 (10)	
C8B	0.1314 (4)	0.2912 (3)	0.4831 (3)	0.0206 (10)	
H8B	0.1373	0.3572	0.4668	0.025*	
C9B	0.0413 (4)	0.2668 (3)	0.4471 (3)	0.0223 (10)	
H9B	0.0323	0.2015	0.4586	0.027*	
C10B	−0.0450 (4)	0.3420 (4)	0.3890 (3)	0.0221 (10)	
C11B	−0.1379 (4)	0.3144 (3)	0.3419 (3)	0.0188 (9)	
C12B	−0.1673 (4)	0.2310 (4)	0.3310 (3)	0.0249 (11)	
C13B	−0.2675 (5)	0.2455 (4)	0.2742 (3)	0.0329 (13)	
H13B	−0.2993	0.1950	0.2603	0.039*	
C14B	−0.3121 (5)	0.3430 (4)	0.2419 (3)	0.0358 (14)	
H14B	−0.3781	0.3675	0.2025	0.043*	

### (I) (2E)-1-(3-Bromothiophen-2-yl)-3-(2-methoxyphenyl)prop-2-en-1-one Atomic displacement parameters (Å^2^)

**Table:**

	*U*^11^	*U*^22^	*U*^33^	*U*^12^	*U*^13^	*U*^23^
Br1C	0.0574 (4)	0.0177 (3)	0.0475 (4)	−0.0001 (3)	0.0161 (3)	−0.0062 (3)
S1C	0.0354 (7)	0.0243 (7)	0.0253 (6)	−0.0110 (5)	0.0106 (5)	−0.0031 (5)
O1C	0.0301 (19)	0.0184 (19)	0.034 (2)	0.0029 (15)	0.0126 (16)	0.0010 (16)
O2C	0.0347 (19)	0.0195 (18)	0.0290 (19)	−0.0002 (16)	0.0118 (16)	0.0054 (15)
C1C	0.038 (3)	0.028 (3)	0.045 (3)	0.002 (3)	0.011 (3)	−0.017 (3)
C2C	0.023 (2)	0.020 (2)	0.015 (2)	−0.0043 (19)	−0.0031 (18)	−0.0017 (18)
C3C	0.025 (2)	0.036 (3)	0.021 (3)	−0.009 (2)	0.000 (2)	−0.006 (2)
C4C	0.043 (3)	0.028 (3)	0.023 (3)	−0.014 (2)	0.002 (2)	0.003 (2)
C5C	0.045 (3)	0.024 (3)	0.041 (4)	−0.008 (3)	0.001 (3)	0.008 (3)
C6C	0.028 (3)	0.019 (3)	0.041 (3)	−0.003 (2)	0.005 (2)	−0.002 (2)
C7C	0.021 (2)	0.019 (2)	0.015 (2)	−0.0024 (19)	−0.0018 (18)	0.0007 (19)
C8C	0.026 (2)	0.015 (2)	0.016 (2)	−0.0005 (19)	−0.0015 (19)	0.0051 (18)
C9C	0.037 (3)	0.013 (2)	0.016 (2)	−0.004 (2)	0.004 (2)	0.0013 (18)
C10C	0.020 (2)	0.022 (3)	0.012 (2)	−0.003 (2)	−0.0016 (18)	−0.0009 (19)
C11C	0.020 (2)	0.023 (2)	0.013 (2)	−0.0047 (19)	0.0022 (18)	−0.0034 (19)
C12C	0.030 (3)	0.025 (3)	0.024 (3)	−0.005 (2)	0.003 (2)	−0.008 (2)
C13C	0.032 (3)	0.046 (4)	0.033 (3)	−0.003 (3)	0.007 (2)	−0.020 (3)
C14C	0.032 (3)	0.039 (3)	0.028 (3)	−0.020 (2)	0.012 (2)	−0.015 (2)
Br1D	0.0565 (4)	0.0175 (3)	0.0486 (4)	−0.0002 (3)	0.0165 (3)	−0.0068 (3)
S1D	0.0340 (7)	0.0239 (6)	0.0238 (6)	−0.0105 (5)	0.0095 (5)	−0.0017 (5)
O1D	0.0310 (19)	0.0191 (19)	0.034 (2)	0.0019 (15)	0.0109 (16)	0.0006 (16)
O2D	0.0306 (18)	0.0201 (19)	0.0296 (19)	0.0050 (15)	0.0082 (15)	0.0054 (15)
C1D	0.027 (3)	0.030 (3)	0.036 (3)	−0.001 (2)	0.013 (2)	−0.011 (2)
C2D	0.023 (2)	0.020 (2)	0.011 (2)	−0.0044 (19)	−0.0006 (18)	−0.0006 (18)
C3D	0.026 (3)	0.033 (3)	0.017 (2)	−0.009 (2)	0.007 (2)	0.004 (2)
C4D	0.040 (3)	0.036 (3)	0.027 (3)	−0.017 (3)	0.007 (2)	0.008 (2)
C5D	0.042 (3)	0.018 (3)	0.037 (3)	−0.002 (2)	0.003 (3)	0.012 (2)
C6D	0.034 (3)	0.017 (3)	0.031 (3)	−0.001 (2)	0.004 (2)	0.005 (2)
C7D	0.022 (2)	0.016 (2)	0.018 (2)	−0.0052 (19)	−0.0002 (19)	−0.0004 (19)
C8D	0.023 (2)	0.021 (2)	0.018 (2)	−0.0030 (19)	0.0023 (18)	−0.0007 (19)
C9D	0.020 (2)	0.011 (2)	0.023 (2)	0.0011 (17)	0.0016 (18)	0.0016 (18)
C10D	0.021 (2)	0.020 (2)	0.015 (2)	−0.0027 (19)	0.0007 (19)	−0.0017 (19)
C11D	0.025 (2)	0.015 (2)	0.015 (2)	−0.0083 (18)	−0.0047 (18)	−0.0002 (18)
C12D	0.026 (2)	0.026 (3)	0.019 (2)	−0.005 (2)	0.000 (2)	−0.007 (2)
C13D	0.029 (3)	0.040 (3)	0.026 (3)	−0.009 (2)	0.011 (2)	−0.014 (2)
C14D	0.033 (3)	0.042 (4)	0.037 (3)	−0.010 (3)	0.016 (2)	−0.017 (3)
Br1A	0.0596 (4)	0.0172 (3)	0.0477 (4)	−0.0055 (3)	−0.0119 (3)	−0.0071 (3)
S1A	0.0270 (6)	0.0241 (6)	0.0251 (7)	0.0023 (5)	−0.0075 (5)	−0.0021 (5)
O1A	0.0327 (19)	0.024 (2)	0.034 (2)	−0.0096 (16)	−0.0082 (16)	−0.0006 (16)
O2A	0.038 (2)	0.0177 (19)	0.0289 (19)	−0.0072 (15)	−0.0100 (16)	0.0035 (15)
C1A	0.040 (3)	0.027 (3)	0.044 (3)	−0.011 (2)	−0.002 (3)	−0.015 (3)
C2A	0.019 (2)	0.020 (2)	0.019 (2)	−0.0024 (19)	0.0033 (18)	−0.0020 (19)
C3A	0.028 (3)	0.035 (3)	0.020 (3)	−0.003 (2)	−0.002 (2)	0.000 (2)
C4A	0.029 (3)	0.031 (3)	0.031 (3)	0.011 (2)	−0.008 (2)	0.008 (2)
C5A	0.038 (3)	0.018 (3)	0.043 (3)	0.001 (2)	−0.002 (3)	0.008 (2)
C6A	0.028 (3)	0.020 (3)	0.042 (3)	−0.004 (2)	−0.007 (2)	0.000 (2)
C7A	0.017 (2)	0.016 (2)	0.017 (2)	−0.0018 (18)	0.0017 (18)	−0.0016 (18)
C8A	0.019 (2)	0.015 (2)	0.021 (2)	−0.0014 (18)	0.0007 (19)	0.0015 (18)
C9A	0.025 (2)	0.017 (2)	0.017 (2)	−0.0041 (19)	−0.0005 (19)	−0.0007 (18)
C10A	0.021 (2)	0.016 (2)	0.017 (2)	−0.0044 (19)	0.0048 (18)	0.0028 (17)
C11A	0.020 (2)	0.020 (2)	0.013 (2)	0.0004 (18)	0.0007 (17)	−0.0014 (17)
C12A	0.029 (3)	0.023 (3)	0.022 (2)	−0.004 (2)	0.002 (2)	−0.006 (2)
C13A	0.029 (3)	0.037 (3)	0.035 (3)	−0.014 (2)	−0.003 (2)	−0.016 (3)
C14A	0.025 (3)	0.044 (4)	0.038 (3)	−0.003 (2)	−0.007 (2)	−0.016 (3)
Br1B	0.0586 (4)	0.0174 (3)	0.0474 (4)	−0.0054 (3)	−0.0120 (3)	−0.0066 (3)
S1B	0.0285 (6)	0.0249 (7)	0.0246 (7)	0.0032 (5)	−0.0073 (5)	−0.0029 (5)
O1B	0.035 (2)	0.0191 (19)	0.034 (2)	−0.0066 (15)	−0.0118 (16)	0.0001 (15)
O2B	0.0334 (19)	0.0164 (18)	0.033 (2)	−0.0056 (15)	−0.0105 (16)	0.0056 (15)
C1B	0.033 (3)	0.032 (3)	0.043 (3)	−0.010 (2)	−0.008 (2)	−0.014 (3)
C2B	0.025 (2)	0.022 (3)	0.013 (2)	−0.005 (2)	0.0034 (18)	−0.0040 (19)
C3B	0.026 (3)	0.032 (3)	0.020 (3)	−0.001 (2)	−0.003 (2)	−0.005 (2)
C4B	0.032 (3)	0.036 (3)	0.027 (3)	0.000 (2)	−0.004 (2)	0.010 (2)
C5B	0.037 (3)	0.023 (3)	0.046 (4)	−0.002 (2)	−0.008 (3)	0.008 (2)
C6B	0.030 (3)	0.020 (3)	0.031 (3)	−0.004 (2)	−0.003 (2)	0.005 (2)
C7B	0.021 (2)	0.019 (2)	0.018 (2)	−0.0038 (19)	0.0042 (19)	−0.0023 (19)
C8B	0.026 (2)	0.011 (2)	0.022 (2)	−0.0033 (19)	0.003 (2)	0.0003 (18)
C9B	0.030 (3)	0.016 (2)	0.018 (2)	−0.004 (2)	−0.001 (2)	0.0004 (18)
C10B	0.027 (2)	0.023 (3)	0.014 (2)	−0.004 (2)	0.0019 (19)	−0.0002 (19)
C11B	0.020 (2)	0.020 (2)	0.015 (2)	−0.0024 (19)	0.0049 (17)	−0.0011 (18)
C12B	0.022 (2)	0.026 (3)	0.025 (3)	0.001 (2)	0.001 (2)	−0.007 (2)
C13B	0.031 (3)	0.041 (3)	0.031 (3)	−0.006 (2)	0.001 (2)	−0.019 (3)
C14B	0.037 (3)	0.037 (3)	0.030 (3)	0.003 (3)	−0.008 (2)	−0.011 (3)

### (I) (2E)-1-(3-Bromothiophen-2-yl)-3-(2-methoxyphenyl)prop-2-en-1-one Geometric parameters (Å, º)

**Table:**

Br1C—C12C	1.880 (5)	Br1A—C12A	1.905 (5)
S1C—C11C	1.721 (5)	S1A—C11A	1.722 (4)
S1C—C14C	1.710 (5)	S1A—C14A	1.705 (5)
O1C—C1C	1.431 (6)	O1A—C1A	1.430 (6)
O1C—C2C	1.348 (6)	O1A—C2A	1.334 (6)
O2C—C10C	1.223 (6)	O2A—C10A	1.226 (6)
C1C—H1CA	0.9800	C1A—H1AA	0.9800
C1C—H1CB	0.9800	C1A—H1AB	0.9800
C1C—H1CC	0.9800	C1A—H1AC	0.9800
C2C—C3C	1.393 (7)	C2A—C3A	1.395 (6)
C2C—C7C	1.418 (6)	C2A—C7A	1.420 (6)
C3C—H3C	0.9500	C3A—H3A	0.9500
C3C—C4C	1.371 (7)	C3A—C4A	1.386 (8)
C4C—H4C	0.9500	C4A—H4A	0.9500
C4C—C5C	1.364 (8)	C4A—C5A	1.399 (8)
C5C—H5C	0.9500	C5A—H5A	0.9500
C5C—C6C	1.395 (7)	C5A—C6A	1.391 (7)
C6C—H6C	0.9500	C6A—H6A	0.9500
C6C—C7C	1.384 (7)	C6A—C7A	1.392 (7)
C7C—C8C	1.460 (6)	C7A—C8A	1.472 (6)
C8C—H8C	0.9500	C8A—H8A	0.9500
C8C—C9C	1.337 (6)	C8A—C9A	1.336 (6)
C9C—H9C	0.9500	C9A—H9A	0.9500
C9C—C10C	1.466 (6)	C9A—C10A	1.466 (6)
C10C—C11C	1.487 (6)	C10A—C11A	1.490 (6)
C11C—C12C	1.379 (7)	C11A—C12A	1.363 (7)
C12C—C13C	1.425 (7)	C12A—C13A	1.401 (7)
C13C—H13C	0.9500	C13A—H13A	0.9500
C13C—C14C	1.351 (8)	C13A—C14A	1.329 (8)
C14C—H14C	0.9500	C14A—H14A	0.9500
Br1D—C12D	1.886 (5)	Br1B—C12B	1.875 (5)
S1D—C11D	1.728 (5)	S1B—C11B	1.742 (5)
S1D—C14D	1.701 (5)	S1B—C14B	1.696 (6)
O1D—C1D	1.433 (5)	O1B—C1B	1.439 (6)
O1D—C2D	1.360 (6)	O1B—C2B	1.353 (6)
O2D—C10D	1.208 (6)	O2B—C10B	1.221 (6)
C1D—H1DA	0.9800	C1B—H1BA	0.9800
C1D—H1DB	0.9800	C1B—H1BB	0.9800
C1D—H1DC	0.9800	C1B—H1BC	0.9800
C2D—C3D	1.400 (6)	C2B—C3B	1.392 (6)
C2D—C7D	1.420 (6)	C2B—C7B	1.414 (6)
C3D—H3D	0.9500	C3B—H3B	0.9500
C3D—C4D	1.385 (8)	C3B—C4B	1.367 (8)
C4D—H4D	0.9500	C4B—H4B	0.9500
C4D—C5D	1.395 (7)	C4B—C5B	1.388 (8)
C5D—H5D	0.9500	C5B—H5B	0.9500
C5D—C6D	1.378 (7)	C5B—C6B	1.387 (7)
C6D—H6D	0.9500	C6B—H6B	0.9500
C6D—C7D	1.401 (7)	C6B—C7B	1.400 (7)
C7D—C8D	1.458 (6)	C7B—C8B	1.449 (6)
C8D—H8D	0.9500	C8B—H8B	0.9500
C8D—C9D	1.319 (6)	C8B—C9B	1.325 (6)
C9D—H9D	0.9500	C9B—H9B	0.9500
C9D—C10D	1.479 (6)	C9B—C10B	1.477 (6)
C10D—C11D	1.484 (6)	C10B—C11B	1.478 (6)
C11D—C12D	1.372 (7)	C11B—C12B	1.368 (7)
C12D—C13D	1.419 (7)	C12B—C13B	1.429 (7)
C13D—H13D	0.9500	C13B—H13B	0.9500
C13D—C14D	1.349 (8)	C13B—C14B	1.387 (8)
C14D—H14D	0.9500	C14B—H14B	0.9500
			
C14C—S1C—C11C	92.4 (2)	C14A—S1A—C11A	91.3 (3)
C2C—O1C—C1C	119.0 (4)	C2A—O1A—C1A	119.2 (4)
O1C—C1C—H1CA	109.5	O1A—C1A—H1AA	109.5
O1C—C1C—H1CB	109.5	O1A—C1A—H1AB	109.5
O1C—C1C—H1CC	109.5	O1A—C1A—H1AC	109.5
H1CA—C1C—H1CB	109.5	H1AA—C1A—H1AB	109.5
H1CA—C1C—H1CC	109.5	H1AA—C1A—H1AC	109.5
H1CB—C1C—H1CC	109.5	H1AB—C1A—H1AC	109.5
O1C—C2C—C3C	125.2 (5)	O1A—C2A—C3A	124.4 (5)
O1C—C2C—C7C	114.9 (4)	O1A—C2A—C7A	116.0 (4)
C3C—C2C—C7C	119.9 (5)	C3A—C2A—C7A	119.7 (5)
C2C—C3C—H3C	119.9	C2A—C3A—H3A	119.9
C4C—C3C—C2C	120.3 (5)	C4A—C3A—C2A	120.1 (5)
C4C—C3C—H3C	119.9	C4A—C3A—H3A	119.9
C3C—C4C—H4C	119.3	C3A—C4A—H4A	119.5
C5C—C4C—C3C	121.4 (5)	C3A—C4A—C5A	121.0 (5)
C5C—C4C—H4C	119.3	C5A—C4A—H4A	119.5
C4C—C5C—H5C	120.6	C4A—C5A—H5A	120.6
C4C—C5C—C6C	118.8 (5)	C6A—C5A—C4A	118.8 (5)
C6C—C5C—H5C	120.6	C6A—C5A—H5A	120.6
C5C—C6C—H6C	118.9	C5A—C6A—H6A	119.2
C7C—C6C—C5C	122.2 (5)	C5A—C6A—C7A	121.5 (5)
C7C—C6C—H6C	118.9	C7A—C6A—H6A	119.2
C2C—C7C—C8C	118.6 (4)	C2A—C7A—C8A	118.0 (4)
C6C—C7C—C2C	117.5 (5)	C6A—C7A—C2A	118.9 (4)
C6C—C7C—C8C	123.9 (5)	C6A—C7A—C8A	123.1 (5)
C7C—C8C—H8C	115.9	C7A—C8A—H8A	116.4
C9C—C8C—C7C	128.2 (5)	C9A—C8A—C7A	127.2 (5)
C9C—C8C—H8C	115.9	C9A—C8A—H8A	116.4
C8C—C9C—H9C	120.2	C8A—C9A—H9A	120.6
C8C—C9C—C10C	119.5 (5)	C8A—C9A—C10A	118.7 (4)
C10C—C9C—H9C	120.2	C10A—C9A—H9A	120.6
O2C—C10C—C9C	121.6 (4)	O2A—C10A—C9A	121.6 (4)
O2C—C10C—C11C	117.8 (4)	O2A—C10A—C11A	117.6 (4)
C9C—C10C—C11C	120.6 (4)	C9A—C10A—C11A	120.8 (4)
C10C—C11C—S1C	113.6 (3)	C10A—C11A—S1A	113.7 (3)
C12C—C11C—S1C	110.2 (4)	C12A—C11A—S1A	109.4 (4)
C12C—C11C—C10C	136.1 (5)	C12A—C11A—C10A	136.9 (4)
C11C—C12C—Br1C	127.4 (4)	C11A—C12A—Br1A	126.4 (4)
C11C—C12C—C13C	113.0 (5)	C11A—C12A—C13A	114.8 (5)
C13C—C12C—Br1C	119.6 (4)	C13A—C12A—Br1A	118.8 (4)
C12C—C13C—H13C	123.9	C12A—C13A—H13A	124.5
C14C—C13C—C12C	112.3 (5)	C14A—C13A—C12A	111.1 (5)
C14C—C13C—H13C	123.9	C14A—C13A—H13A	124.5
S1C—C14C—H14C	123.9	S1A—C14A—H14A	123.2
C13C—C14C—S1C	112.2 (4)	C13A—C14A—S1A	113.5 (4)
C13C—C14C—H14C	123.9	C13A—C14A—H14A	123.2
C14D—S1D—C11D	92.0 (3)	C14B—S1B—C11B	92.8 (3)
C2D—O1D—C1D	119.1 (4)	C2B—O1B—C1B	119.4 (4)
O1D—C1D—H1DA	109.5	O1B—C1B—H1BA	109.5
O1D—C1D—H1DB	109.5	O1B—C1B—H1BB	109.5
O1D—C1D—H1DC	109.5	O1B—C1B—H1BC	109.5
H1DA—C1D—H1DB	109.5	H1BA—C1B—H1BB	109.5
H1DA—C1D—H1DC	109.5	H1BA—C1B—H1BC	109.5
H1DB—C1D—H1DC	109.5	H1BB—C1B—H1BC	109.5
O1D—C2D—C3D	123.7 (5)	O1B—C2B—C3B	124.8 (5)
O1D—C2D—C7D	115.7 (4)	O1B—C2B—C7B	115.1 (4)
C3D—C2D—C7D	120.6 (5)	C3B—C2B—C7B	120.1 (5)
C2D—C3D—H3D	120.6	C2B—C3B—H3B	119.9
C4D—C3D—C2D	118.8 (5)	C4B—C3B—C2B	120.3 (5)
C4D—C3D—H3D	120.6	C4B—C3B—H3B	119.9
C3D—C4D—H4D	119.4	C3B—C4B—H4B	119.4
C3D—C4D—C5D	121.2 (5)	C3B—C4B—C5B	121.2 (5)
C5D—C4D—H4D	119.4	C5B—C4B—H4B	119.4
C4D—C5D—H5D	120.0	C4B—C5B—H5B	120.5
C6D—C5D—C4D	120.0 (5)	C6B—C5B—C4B	118.9 (6)
C6D—C5D—H5D	120.0	C6B—C5B—H5B	120.6
C5D—C6D—H6D	119.6	C5B—C6B—H6B	119.2
C5D—C6D—C7D	120.7 (5)	C5B—C6B—C7B	121.6 (5)
C7D—C6D—H6D	119.6	C7B—C6B—H6B	119.2
C2D—C7D—C8D	118.8 (4)	C2B—C7B—C8B	119.2 (5)
C6D—C7D—C2D	118.5 (4)	C6B—C7B—C2B	117.9 (5)
C6D—C7D—C8D	122.7 (5)	C6B—C7B—C8B	122.9 (5)
C7D—C8D—H8D	115.7	C7B—C8B—H8B	115.7
C9D—C8D—C7D	128.6 (5)	C9B—C8B—C7B	128.6 (5)
C9D—C8D—H8D	115.7	C9B—C8B—H8B	115.7
C8D—C9D—H9D	120.1	C8B—C9B—H9B	120.2
C8D—C9D—C10D	119.8 (4)	C8B—C9B—C10B	119.6 (4)
C10D—C9D—H9D	120.1	C10B—C9B—H9B	120.2
O2D—C10D—C9D	122.1 (4)	O2B—C10B—C9B	121.9 (5)
O2D—C10D—C11D	117.5 (4)	O2B—C10B—C11B	118.1 (4)
C9D—C10D—C11D	120.3 (4)	C9B—C10B—C11B	120.0 (4)
C10D—C11D—S1D	113.3 (3)	C10B—C11B—S1B	113.0 (4)
C12D—C11D—S1D	109.7 (4)	C12B—C11B—S1B	109.7 (4)
C12D—C11D—C10D	137.0 (5)	C12B—C11B—C10B	137.3 (5)
C11D—C12D—Br1D	127.0 (4)	C11B—C12B—Br1B	127.1 (4)
C11D—C12D—C13D	113.9 (5)	C11B—C12B—C13B	114.3 (5)
C13D—C12D—Br1D	119.0 (4)	C13B—C12B—Br1B	118.6 (4)
C12D—C13D—H13D	124.3	C12B—C13B—H13B	124.5
C14D—C13D—C12D	111.4 (5)	C14B—C13B—C12B	111.0 (5)
C14D—C13D—H13D	124.3	C14B—C13B—H13B	124.5
S1D—C14D—H14D	123.5	S1B—C14B—H14B	123.9
C13D—C14D—S1D	113.0 (4)	C13B—C14B—S1B	112.2 (4)
C13D—C14D—H14D	123.5	C13B—C14B—H14B	123.9
			
Br1C—C12C—C13C—C14C	−179.2 (4)	Br1A—C12A—C13A—C14A	−179.2 (4)
S1C—C11C—C12C—Br1C	178.8 (3)	S1A—C11A—C12A—Br1A	178.5 (3)
S1C—C11C—C12C—C13C	−0.3 (6)	S1A—C11A—C12A—C13A	−0.3 (6)
O1C—C2C—C3C—C4C	179.4 (5)	O1A—C2A—C3A—C4A	179.9 (5)
O1C—C2C—C7C—C6C	−179.2 (5)	O1A—C2A—C7A—C6A	−179.4 (5)
O1C—C2C—C7C—C8C	1.0 (7)	O1A—C2A—C7A—C8A	0.1 (6)
O2C—C10C—C11C—S1C	−2.3 (6)	O2A—C10A—C11A—S1A	−2.4 (6)
O2C—C10C—C11C—C12C	174.3 (5)	O2A—C10A—C11A—C12A	174.3 (5)
C1C—O1C—C2C—C3C	−1.7 (8)	C1A—O1A—C2A—C3A	−3.4 (7)
C1C—O1C—C2C—C7C	177.2 (4)	C1A—O1A—C2A—C7A	177.4 (4)
C2C—C3C—C4C—C5C	−1.3 (9)	C2A—C3A—C4A—C5A	−0.5 (9)
C2C—C7C—C8C—C9C	−178.1 (5)	C2A—C7A—C8A—C9A	−176.5 (4)
C3C—C2C—C7C—C6C	−0.2 (7)	C3A—C2A—C7A—C6A	1.3 (7)
C3C—C2C—C7C—C8C	179.9 (4)	C3A—C2A—C7A—C8A	−179.2 (4)
C3C—C4C—C5C—C6C	1.6 (9)	C3A—C4A—C5A—C6A	1.4 (9)
C4C—C5C—C6C—C7C	−1.3 (9)	C4A—C5A—C6A—C7A	−0.9 (9)
C5C—C6C—C7C—C2C	0.6 (8)	C5A—C6A—C7A—C2A	−0.4 (8)
C5C—C6C—C7C—C8C	−179.5 (5)	C5A—C6A—C7A—C8A	−179.9 (5)
C6C—C7C—C8C—C9C	2.1 (9)	C6A—C7A—C8A—C9A	3.0 (8)
C7C—C2C—C3C—C4C	0.5 (8)	C7A—C2A—C3A—C4A	−0.9 (8)
C7C—C8C—C9C—C10C	177.3 (4)	C7A—C8A—C9A—C10A	176.8 (4)
C8C—C9C—C10C—O2C	−7.8 (8)	C8A—C9A—C10A—O2A	−8.6 (7)
C8C—C9C—C10C—C11C	172.9 (4)	C8A—C9A—C10A—C11A	171.6 (4)
C9C—C10C—C11C—S1C	177.0 (4)	C9A—C10A—C11A—S1A	177.4 (3)
C9C—C10C—C11C—C12C	−6.4 (8)	C9A—C10A—C11A—C12A	−5.9 (8)
C10C—C11C—C12C—Br1C	2.1 (9)	C10A—C11A—C12A—Br1A	1.7 (9)
C10C—C11C—C12C—C13C	−176.9 (5)	C10A—C11A—C12A—C13A	−177.0 (5)
C11C—S1C—C14C—C13C	−0.5 (5)	C11A—S1A—C14A—C13A	−0.8 (5)
C11C—C12C—C13C—C14C	−0.1 (7)	C11A—C12A—C13A—C14A	−0.4 (7)
C12C—C13C—C14C—S1C	0.4 (6)	C12A—C13A—C14A—S1A	0.8 (7)
C14C—S1C—C11C—C10C	177.9 (3)	C14A—S1A—C11A—C10A	178.2 (4)
C14C—S1C—C11C—C12C	0.5 (4)	C14A—S1A—C11A—C12A	0.6 (4)
Br1D—C12D—C13D—C14D	−177.7 (4)	Br1B—C12B—C13B—C14B	−178.6 (4)
S1D—C11D—C12D—Br1D	178.3 (3)	S1B—C11B—C12B—Br1B	179.0 (3)
S1D—C11D—C12D—C13D	2.3 (5)	S1B—C11B—C12B—C13B	0.0 (5)
O1D—C2D—C3D—C4D	−178.4 (5)	O1B—C2B—C3B—C4B	178.9 (5)
O1D—C2D—C7D—C6D	−179.6 (5)	O1B—C2B—C7B—C6B	−179.0 (4)
O1D—C2D—C7D—C8D	−0.1 (7)	O1B—C2B—C7B—C8B	0.6 (7)
O2D—C10D—C11D—S1D	−3.4 (6)	O2B—C10B—C11B—S1B	−1.9 (6)
O2D—C10D—C11D—C12D	176.5 (5)	O2B—C10B—C11B—C12B	175.8 (5)
C1D—O1D—C2D—C3D	−2.2 (8)	C1B—O1B—C2B—C3B	−1.4 (7)
C1D—O1D—C2D—C7D	178.0 (4)	C1B—O1B—C2B—C7B	178.4 (4)
C2D—C3D—C4D—C5D	−3.9 (9)	C2B—C3B—C4B—C5B	1.0 (9)
C2D—C7D—C8D—C9D	−177.0 (5)	C2B—C7B—C8B—C9B	−178.6 (5)
C3D—C2D—C7D—C6D	0.6 (7)	C3B—C2B—C7B—C6B	0.9 (7)
C3D—C2D—C7D—C8D	−179.9 (5)	C3B—C2B—C7B—C8B	−179.6 (4)
C3D—C4D—C5D—C6D	4.5 (9)	C3B—C4B—C5B—C6B	−1.1 (9)
C4D—C5D—C6D—C7D	−2.5 (9)	C4B—C5B—C6B—C7B	1.0 (9)
C5D—C6D—C7D—C2D	−0.1 (8)	C5B—C6B—C7B—C2B	−0.9 (8)
C5D—C6D—C7D—C8D	−179.6 (5)	C5B—C6B—C7B—C8B	179.5 (5)
C6D—C7D—C8D—C9D	2.5 (9)	C6B—C7B—C8B—C9B	0.9 (8)
C7D—C2D—C3D—C4D	1.3 (8)	C7B—C2B—C3B—C4B	−0.9 (8)
C7D—C8D—C9D—C10D	176.3 (4)	C7B—C8B—C9B—C10B	177.1 (4)
C8D—C9D—C10D—O2D	−7.7 (8)	C8B—C9B—C10B—O2B	−7.7 (7)
C8D—C9D—C10D—C11D	171.6 (4)	C8B—C9B—C10B—C11B	173.6 (4)
C9D—C10D—C11D—S1D	177.2 (3)	C9B—C10B—C11B—S1B	176.9 (3)
C9D—C10D—C11D—C12D	−2.9 (8)	C9B—C10B—C11B—C12B	−5.4 (9)
C10D—C11D—C12D—Br1D	−1.6 (8)	C10B—C11B—C12B—Br1B	1.2 (9)
C10D—C11D—C12D—C13D	−177.6 (5)	C10B—C11B—C12B—C13B	−177.8 (5)
C11D—S1D—C14D—C13D	1.4 (5)	C11B—S1B—C14B—C13B	0.7 (5)
C11D—C12D—C13D—C14D	−1.3 (7)	C11B—C12B—C13B—C14B	0.5 (7)
C12D—C13D—C14D—S1D	−0.3 (7)	C12B—C13B—C14B—S1B	−0.8 (6)
C14D—S1D—C11D—C10D	177.8 (4)	C14B—S1B—C11B—C10B	178.0 (4)
C14D—S1D—C11D—C12D	−2.1 (4)	C14B—S1B—C11B—C12B	−0.4 (4)

### (I) (2E)-1-(3-Bromothiophen-2-yl)-3-(2-methoxyphenyl)prop-2-en-1-one Hydrogen-bond geometry (Å, º)

**Table:**

*D*—H···*A*	*D*—H	H···*A*	*D*···*A*	*D*—H···*A*
C9*C*—H9*C*···Br1*C*	0.95	2.68	3.401 (5)	133
C9*D*—H9*D*···Br1*D*	0.95	2.69	3.405 (4)	133
C9*A*—H9*A*···Br1*A*	0.95	2.69	3.398 (5)	132
C9*B*—H9*B*···Br1*B*	0.95	2.68	3.401 (5)	133

### (II) (2E)-1-(3-Bromothiophen-2-yl)-3-(3,4-dimethoxyphenyl)prop-2-en-1-one Crystal data

**Table:**

C_15_H_13_BrO_3_S	*F*(000) = 1424
*M**_r_* = 353.22	*D*_x_ = 1.664 Mg m^−^^3^
Monoclinic, *I*2/*a*	Cu *K*α radiation, λ = 1.54184 Å
*a* = 13.4748 (7) Å	Cell parameters from 2804 reflections
*b* = 8.3853 (3) Å	θ = 5.5–71.4°
*c* = 25.0214 (9) Å	µ = 5.40 mm^−^^1^
β = 93.957 (4)°	*T* = 173 K
*V* = 2820.4 (2) Å^3^	Irregular, yellow
*Z* = 8	0.32 × 0.28 × 0.22 mm

### (II) (2E)-1-(3-Bromothiophen-2-yl)-3-(3,4-dimethoxyphenyl)prop-2-en-1-one Data collection

**Table:**

Agilent Eos Gemini diffractometer	2690 independent reflections
Radiation source: Enhance (Cu) X-ray Source	2399 reflections with *I* > 2σ(*I*)
Detector resolution: 16.0416 pixels mm^-1^	*R*_int_ = 0.026
ω scans	θ_max_ = 71.4°, θ_min_ = 3.5°
Absorption correction: multi-scan (CrysAlis PRO; Agilent, 2014)	*h* = −13→16
*T*_min_ = 0.726, *T*_max_ = 1.000	*k* = −7→10
5523 measured reflections	*l* = −30→23

### (II) (2E)-1-(3-Bromothiophen-2-yl)-3-(3,4-dimethoxyphenyl)prop-2-en-1-one Refinement

**Table:**

Refinement on *F*^2^	Primary atom site location: structure-invariant direct methods
Least-squares matrix: full	Hydrogen site location: inferred from neighbouring sites
*R*[*F*^2^ > 2σ(*F*^2^)] = 0.074	H-atom parameters constrained
*wR*(*F*^2^) = 0.187	*w* = 1/[σ^2^(*F*_o_^2^) + (0.0864*P*)^2^ + 41.1386*P*] where *P* = (*F*_o_^2^ + 2*F*_c_^2^)/3
*S* = 1.04	(Δ/σ)_max_ < 0.001
2690 reflections	Δρ_max_ = 3.38 e Å^−^^3^
183 parameters	Δρ_min_ = −2.20 e Å^−^^3^
0 restraints	

### (II) (2E)-1-(3-Bromothiophen-2-yl)-3-(3,4-dimethoxyphenyl)prop-2-en-1-one Special details

**Table:**

Experimental. Absorption correction: CrysAlisPro, Agilent Technologies, Version 1.171.37.31 (release 14-01-2014 CrysAlis171 .NET) (compiled Jan 14 2014,18:38:05) Empirical absorption correction using spherical harmonics, implemented in SCALE3 ABSPACK scaling algorithm
Geometry. All e.s.d.'s (except the e.s.d. in the dihedral angle between two l.s. planes) are estimated using the full covariance matrix. The cell e.s.d.'s are taken into account individually in the estimation of e.s.d.'s in distances, angles and torsion angles; correlations between e.s.d.'s in cell parameters are only used when they are defined by crystal symmetry. An approximate (isotropic) treatment of cell e.s.d.'s is used for estimating e.s.d.'s involving l.s. planes.

### (II) (2E)-1-(3-Bromothiophen-2-yl)-3-(3,4-dimethoxyphenyl)prop-2-en-1-one Fractional atomic coordinates and isotropic or equivalent isotropic displacement parameters (Å^2^)

**Table:**

	*x*	*y*	*z*	*U*_iso_*/*U*_eq_	
Br1	0.61968 (8)	0.91781 (8)	0.53629 (3)	0.0580 (3)	
S1	0.64474 (11)	0.66323 (16)	0.68963 (5)	0.0276 (3)	
O1	0.6334 (3)	0.3966 (4)	0.61923 (14)	0.0274 (8)	
O2	0.5945 (3)	0.6136 (4)	0.32362 (14)	0.0240 (8)	
O3	0.6189 (3)	0.3395 (4)	0.28114 (13)	0.0240 (8)	
C1	0.6314 (3)	0.5174 (6)	0.59246 (18)	0.0182 (9)	
C2	0.6360 (3)	0.6745 (6)	0.62051 (18)	0.0177 (9)	
C3	0.6336 (4)	0.8322 (6)	0.60615 (19)	0.0218 (10)	
C4	0.6394 (4)	0.9416 (7)	0.6490 (2)	0.0272 (11)	
H4	0.6388	1.0542	0.6449	0.033*	
C5	0.6460 (4)	0.8652 (7)	0.6967 (2)	0.0296 (12)	
H5	0.6508	0.9182	0.7304	0.035*	
C6	0.6245 (4)	0.5171 (6)	0.53382 (19)	0.0235 (10)	
H6	0.6173	0.6159	0.5153	0.028*	
C7	0.6282 (4)	0.3831 (6)	0.50554 (19)	0.0204 (10)	
H7	0.6344	0.2862	0.5252	0.025*	
C8	0.6235 (4)	0.3708 (6)	0.44730 (19)	0.0191 (9)	
C9	0.6359 (4)	0.2233 (6)	0.4232 (2)	0.0233 (10)	
H9	0.6460	0.1312	0.4450	0.028*	
C10	0.6339 (4)	0.2082 (6)	0.3679 (2)	0.0226 (10)	
H10	0.6417	0.1060	0.3523	0.027*	
C11	0.6207 (4)	0.3397 (6)	0.33556 (18)	0.0185 (9)	
C12	0.6083 (3)	0.4915 (5)	0.35916 (19)	0.0173 (9)	
C13	0.6105 (3)	0.5049 (6)	0.41371 (19)	0.0182 (9)	
H13	0.6031	0.6071	0.4293	0.022*	
C14	0.6252 (4)	0.1871 (6)	0.2557 (2)	0.0280 (11)	
H14A	0.6236	0.2016	0.2168	0.042*	
H14B	0.5688	0.1208	0.2647	0.042*	
H14C	0.6876	0.1347	0.2682	0.042*	
C15	0.5831 (5)	0.7695 (6)	0.3449 (2)	0.0299 (12)	
H15A	0.5705	0.8455	0.3155	0.045*	
H15B	0.6440	0.7999	0.3661	0.045*	
H15C	0.5269	0.7704	0.3678	0.045*	

### (II) (2E)-1-(3-Bromothiophen-2-yl)-3-(3,4-dimethoxyphenyl)prop-2-en-1-one Atomic displacement parameters (Å^2^)

**Table:**

	*U*^11^	*U*^22^	*U*^33^	*U*^12^	*U*^13^	*U*^23^
Br1	0.1252 (8)	0.0253 (4)	0.0221 (4)	−0.0140 (4)	−0.0044 (4)	0.0097 (2)
S1	0.0458 (8)	0.0257 (7)	0.0108 (6)	−0.0012 (5)	−0.0015 (5)	0.0002 (5)
O1	0.044 (2)	0.0208 (18)	0.0174 (17)	−0.0012 (16)	0.0004 (15)	0.0032 (14)
O2	0.044 (2)	0.0126 (16)	0.0151 (17)	0.0035 (15)	0.0004 (14)	0.0028 (13)
O3	0.045 (2)	0.0161 (17)	0.0111 (16)	0.0013 (15)	0.0015 (14)	−0.0022 (13)
C1	0.020 (2)	0.020 (2)	0.015 (2)	0.0015 (18)	−0.0009 (17)	0.0015 (18)
C2	0.020 (2)	0.021 (2)	0.011 (2)	−0.0017 (18)	−0.0024 (16)	0.0006 (18)
C3	0.030 (2)	0.022 (2)	0.014 (2)	−0.005 (2)	0.0001 (18)	0.0026 (19)
C4	0.033 (3)	0.021 (2)	0.028 (3)	−0.002 (2)	0.001 (2)	−0.006 (2)
C5	0.032 (3)	0.032 (3)	0.024 (3)	−0.001 (2)	−0.001 (2)	−0.011 (2)
C6	0.036 (3)	0.019 (2)	0.015 (2)	−0.001 (2)	−0.0031 (19)	0.0007 (18)
C7	0.026 (2)	0.019 (2)	0.015 (2)	−0.0017 (19)	−0.0029 (18)	0.0020 (18)
C8	0.025 (2)	0.019 (2)	0.013 (2)	−0.0018 (19)	−0.0029 (17)	−0.0012 (18)
C9	0.038 (3)	0.012 (2)	0.019 (2)	0.000 (2)	0.001 (2)	0.0038 (18)
C10	0.034 (3)	0.013 (2)	0.020 (2)	0.0013 (19)	−0.0009 (19)	−0.0029 (19)
C11	0.026 (2)	0.015 (2)	0.014 (2)	0.0001 (18)	0.0000 (17)	−0.0025 (18)
C12	0.021 (2)	0.012 (2)	0.018 (2)	0.0017 (17)	−0.0006 (16)	0.0016 (18)
C13	0.024 (2)	0.013 (2)	0.017 (2)	0.0008 (18)	−0.0012 (17)	−0.0034 (18)
C14	0.046 (3)	0.021 (3)	0.017 (2)	0.000 (2)	0.000 (2)	−0.007 (2)
C15	0.048 (3)	0.012 (2)	0.029 (3)	0.001 (2)	−0.002 (2)	0.002 (2)

### (II) (2E)-1-(3-Bromothiophen-2-yl)-3-(3,4-dimethoxyphenyl)prop-2-en-1-one Geometric parameters (Å, º)

**Table:**

Br1—C3	1.887 (5)	C7—H7	0.9500
S1—C2	1.728 (5)	C7—C8	1.458 (7)
S1—C5	1.703 (6)	C8—C9	1.391 (7)
O1—C1	1.213 (6)	C8—C13	1.407 (7)
O2—C12	1.360 (6)	C9—H9	0.9500
O2—C15	1.424 (6)	C9—C10	1.388 (7)
O3—C11	1.360 (6)	C10—H10	0.9500
O3—C14	1.433 (6)	C10—C11	1.372 (7)
C1—C2	1.492 (7)	C11—C12	1.417 (6)
C1—C6	1.464 (6)	C12—C13	1.368 (7)
C2—C3	1.370 (7)	C13—H13	0.9500
C3—C4	1.409 (7)	C14—H14A	0.9800
C4—H4	0.9500	C14—H14B	0.9800
C4—C5	1.353 (8)	C14—H14C	0.9800
C5—H5	0.9500	C15—H15A	0.9800
C6—H6	0.9500	C15—H15B	0.9800
C6—C7	1.331 (7)	C15—H15C	0.9800
			
C5—S1—C2	92.8 (3)	C8—C9—H9	119.4
C12—O2—C15	117.4 (4)	C10—C9—C8	121.2 (4)
C11—O3—C14	116.7 (4)	C10—C9—H9	119.4
O1—C1—C2	118.6 (4)	C9—C10—H10	119.7
O1—C1—C6	123.3 (5)	C11—C10—C9	120.5 (4)
C6—C1—C2	118.0 (4)	C11—C10—H10	119.7
C1—C2—S1	114.8 (3)	O3—C11—C10	125.6 (4)
C3—C2—S1	108.3 (4)	O3—C11—C12	115.1 (4)
C3—C2—C1	136.8 (4)	C10—C11—C12	119.3 (4)
C2—C3—Br1	127.5 (4)	O2—C12—C11	114.7 (4)
C2—C3—C4	115.4 (5)	O2—C12—C13	125.6 (4)
C4—C3—Br1	117.0 (4)	C13—C12—C11	119.6 (4)
C3—C4—H4	124.4	C8—C13—H13	119.2
C5—C4—C3	111.2 (5)	C12—C13—C8	121.6 (4)
C5—C4—H4	124.4	C12—C13—H13	119.2
S1—C5—H5	123.9	O3—C14—H14A	109.5
C4—C5—S1	112.2 (4)	O3—C14—H14B	109.5
C4—C5—H5	123.9	O3—C14—H14C	109.5
C1—C6—H6	118.9	H14A—C14—H14B	109.5
C7—C6—C1	122.1 (5)	H14A—C14—H14C	109.5
C7—C6—H6	118.9	H14B—C14—H14C	109.5
C6—C7—H7	116.9	O2—C15—H15A	109.5
C6—C7—C8	126.2 (5)	O2—C15—H15B	109.5
C8—C7—H7	116.9	O2—C15—H15C	109.5
C9—C8—C7	119.8 (4)	H15A—C15—H15B	109.5
C9—C8—C13	117.7 (4)	H15A—C15—H15C	109.5
C13—C8—C7	122.4 (4)	H15B—C15—H15C	109.5
			
Br1—C3—C4—C5	178.1 (4)	C6—C1—C2—S1	−179.6 (4)
S1—C2—C3—Br1	−177.5 (3)	C6—C1—C2—C3	1.8 (8)
S1—C2—C3—C4	0.6 (6)	C6—C7—C8—C9	175.0 (5)
O1—C1—C2—S1	0.3 (6)	C6—C7—C8—C13	−2.4 (8)
O1—C1—C2—C3	−178.2 (6)	C7—C8—C9—C10	−178.7 (5)
O1—C1—C6—C7	−5.5 (8)	C7—C8—C13—C12	178.6 (4)
O2—C12—C13—C8	178.8 (4)	C8—C9—C10—C11	0.8 (8)
O3—C11—C12—O2	1.3 (6)	C9—C8—C13—C12	1.1 (7)
O3—C11—C12—C13	−179.0 (4)	C9—C10—C11—O3	179.0 (5)
C1—C2—C3—Br1	1.1 (9)	C9—C10—C11—C12	−0.5 (8)
C1—C2—C3—C4	179.2 (5)	C10—C11—C12—O2	−179.2 (4)
C1—C6—C7—C8	−179.1 (5)	C10—C11—C12—C13	0.5 (7)
C2—S1—C5—C4	0.5 (5)	C11—C12—C13—C8	−0.8 (7)
C2—C1—C6—C7	174.4 (5)	C13—C8—C9—C10	−1.1 (8)
C2—C3—C4—C5	−0.2 (7)	C14—O3—C11—C10	4.1 (7)
C3—C4—C5—S1	−0.2 (6)	C14—O3—C11—C12	−176.4 (4)
C5—S1—C2—C1	−179.5 (4)	C15—O2—C12—C11	−179.1 (4)
C5—S1—C2—C3	−0.6 (4)	C15—O2—C12—C13	1.3 (7)

### (II) (2E)-1-(3-Bromothiophen-2-yl)-3-(3,4-dimethoxyphenyl)prop-2-en-1-one Hydrogen-bond geometry (Å, º)

**Table:**

*D*—H···*A*	*D*—H	H···*A*	*D*···*A*	*D*—H···*A*
C5—H5···O2^i^	0.95	2.52	3.301 (6)	140
C5—H5···O3^i^	0.95	2.45	3.291 (6)	148
C6—H6···Br1	0.95	2.59	3.361 (5)	139
C14—H14*A*···O1^ii^	0.98	2.59	3.495 (6)	154

Symmetry codes: (i) *x*, −*y*+3/2, *z*+1/2; (ii) *x*, −*y*+1/2, *z*−1/2.
